# Physiological and transcriptome analysis of *Candida albicans* in response to X33 antimicrobial oligopeptide treatment

**DOI:** 10.3389/fcimb.2023.1123393

**Published:** 2023-01-19

**Authors:** Qunlin Lu, Yuanxiu Wang, Xing Liao, Fu Zhou, Bin Zhang, Xiaoyu Wu

**Affiliations:** ^1^ College of Bioscience and Bioengineering, Jiangxi Agriculture University, Nanchang, China; ^2^ Jiangxi Engineering Laboratory for the Development and Utilization of Agricultural Microbial Resources, Jiangxi Agriculture University, Nanchang, China; ^3^ Collaborative Innovation Center of Postharvest Key Technology and Quality Safety of Fruits and Vegetables in Jiangxi Province, Jiangxi Agriculture University, Nanchang, China

**Keywords:** X33 antimicrobial oligopeptide, *Candida albicans*, antimicrobial mechanism, transcriptomics, cell damage

## Abstract

**Introduction:**

*Candida albicans* is an opportunistic pathogenic fungus, which frequently causes systemic or local fungal infections in *humans*. The evolution of its drug-resistant mutants necessitate an urgent development of novel antimicrobial agents.

**Results:**

Here, we explored the antimicrobial activity and inhibitory mechanisms of X33 antimicrobial oligopeptide (X33 AMOP) against *C. albicans*. The oxford cup test results showed that X33 AMOP had strong inhibitory activity against *C. albicans*, and its MIC and MFC were 0.625 g/L and 2.5 g/L, respectively. Moreover, SEM and TEM showed that X33 AMOP disrupted the integrity of cell membrane. The AKP, ROS, H_2_O_2_ and MDA contents increased, while the reducing sugar, soluble protein, and pyruvate contents decreased after the X33 AMOP treatment. This indicated that X33 AMOP could damage the mitochondrial integrity of the cells, thereby disrupting the energy metabolism by inducing oxidative stress in *C. albicans*. Furthermore, transcriptome analysis showed that X33 AMOP treatment resulted in the differential expression of 1140 genes, among which 532 were up-regulated, and 608 were down-regulated. These DEGs were related to protein, nucleic acid, and carbohydrate metabolism, and their expression changes were consistent with the changes in physiological characteristics. Moreover, we found that X33 AMOP could effectively inhibit the virulence attributes of *C. albicans* by reducing phospholipase activity and disrupting hypha formation.

**Discussion:**

These findings provide the first-ever detailed reference for the inhibitory mechanisms of X33 AMOP against *C. albicans* and suggest that X33 AMOP is a potential drug candidate for treating *C. albicans* infections.

## Highlights

- X33 AMOP have a negative impact on the growth of C. albicans.
- X33 AMOP suppressed morphological transformation of C. albicans.
- X33 AMOP interfered with the intracellular proteins and reducing sugar synthesis of C. albicans.
- X33 AMOP caused genetic information delivery change, destruction of cell wall and cell membrane integrity.
- X33 AMOP accelerated the production of ROS, MDA and H2O2, and induced oxidative damage.

## 1 Introduction

Fungal infections have become serious threats to human health, with a mortality rate of up to 45% (Rauseo et al., 2020). *C. albicans* is an opportunistic human fungal pathogen, which can change from yeast to hyphal growth forms and is widely distributed on the skin and the mucous membrane of the oral cavity and vagina (Ghannoum et al., 2010). Though a human commensal, *C. albicans* infects the body when the host’s immunity is reduced, causing superficial and invasive infections (Ruhnke, 2006). In extreme cases, invasive candidiasis causes a serious health threat to patients (Dimopoulos et al., 2007). Among the *Candida* species, *C. albicans* is the most widely distributed species, causing life-threatening systemic blood infections in immunocompromised humans. For example, candidemia caused by *C. albicans*, has been identified as the third or fourth most cause of bloodstream infections and the first cause of severe infections in the intensive care units (Pappas et al., 2016). *C. albicans* invasive infections frequently result in candidemia and invasive candidiasis, leading to 20 - 34% and 45 - 70% of mortality rates in newborns and adults, respectively (Antinori et al., 2016; Fu et al., 2017). Currently, various drugs, including polyenes (amphotericin B, nystatin), azoles (fluconazole, itraconazole), echinocandins (caspofungin, micafungin), etc., have been discovered and used as clinical treatment of various fungal infections. Despite exerting a bacteriostatic effect against *C. albicans* by destroying its cell wall or cell membrane (Hoehamer et al., 2010), these drugs are toxic to the liver and kidney, expensive, and cannot contain the drug-resistant strains. Therefore, developing novel antimicrobial agents that are effective and safe is crucial for treating fungal infections.

Antimicrobial peptides (AMPs) are 10 - 50 amino acid residues containing functional peptides with defensive activities for human immunity. Thus, AMPs are considered promising drugs due to their antibacterial, antifungal, antivirus, and antitumor properties (Huan et al., 2020). AMPs are widely produced by prokaryotes, unicellular bacteria (non-filamentous bacteria), and eukaryotic fungi capable of protecting their host from pathogens. *Streptomyces* produce various products with important biological activities, which can serve as antimicrobial peptides, antibiotics, immunosuppressants, anticancer drugs, etc., (Berdy, 2012). Notably, secondary metabolites produced by *Streptomyces* play a key role in treating infectious diseases caused by *C. albicans* (Sivalingam et al., 2019). For example, ϵ-polylysine, produced by *Streptomyces albus*, exerts a wide range of antimicrobial activities against yeast and is widely applied in food, medical, and biopharmaceutical industries (Shi et al., 2015). Moreover, *Streptomyces lavendulae* X33 (isolated in our previous study) secretes X33 antimicrobial oligopeptide (X33 AMOP), which strongly inhibits *Penicillium digitatum* (Lin et al., 2020). The recent advancement in transcriptomic analysis has greatly enhanced the ability to analyze the inhibition mechanisms of antimicrobial peptides against pathogens. For example, transcriptome analysis has been used to explore the changes in *C. albicans* target areas after treatment with MAF-1 (Wang et al., 2017).

In this study, we found that X33 AMOP also exhibits preferable antimicrobial effects on *C. albicans*. We used transcriptome and physiological analysis methods to investigate the bacteriostatic mechanism of X33 AMOP against *C. albicans.* The findings of this study provide a theoretical basis for antifungal infection drug research and development.

## 2 Materials and methods

### 2.1 Strains and reagents

According to the previous method, X33 AMOP was isolated from the fermentation supernatant of *S. lavendulae* X33 (stored in the typical culture preservation center of China, No. CCTCC M2013163) (Lin et al., 2020). *C. albicans* ATCC 12322 was obtained from Jiangxi microbial strain collection and management center. *C. albicans* was stored in glycerol at - 80℃, and then streaked onto YPD agar (1% yeast extract, 2% peptone, 2% dextrose, and 2% agar). A single colony was then inoculated in YPD broth (1% yeast extract, 2% peptone, and 2% dextrose) to obtain fresh yeast cells. The cells were resuspended with RPMI 1640 and adjusted to 10^7^ CFU/mL to prepare cell suspension for further analysis.

### 2.2 Evaluation of X33 AMOP antifungal activity

To evaluate the inhibitory effect of X33 AMOP on the growth of *C. albicans*, the minimum inhibitory concentration (MIC) of X33 AMOP was determined according to a previous method with slight modification (Sun et al., 2022). The concentration of X33 AMOP was half diluted from 20 g/L to 0.039 g/L by continuous dilution method, and sterile water was used as a control.

The activity of the X33 AMOP against *C. albicans* was tested using the oxford cup method. Briefly, the cell suspension was evenly spread on the YPD agar under sterile conditions, and the oxford cups were placed in the center of the medium. Different concentrations of X33 AMOP (1/4 MIC, 1/2 MIC and MIC) and sterile water (control) were added (200 µL each) to each cup, and plates were subsequently cultivated at 37℃ for 24 h. The diameter of the antibacterial circle was measured with a vernier caliper using the cross method and was stated as mean ± standard error. These tests were repeated three independent times.

The time sterilization curve of X33 AMOP against *C. albicans* was drawn by sampling at different time points. The X33 AMOP with different concentrations were added, and sterile water as the control. The conical flasks were incubated at 37℃ with 200 r/min shaking to 12 h. Fermentation broth was taken at 2 h intervals to measure the absorbance. The growth curve was plotted as absorbance at 560 nm against time interval.

### 2.3 RNA sequencing and RT-PCR

The cells were added to YPD broth and cultured at 37℃ for 4 h with X33 AMOP at 0.625 g/L for the E group and 0 g/L for the C group. Thereafter, cells were harvested, washed several times with sterile water and quickly frozen in liquid nitrogen for total RNA extraction. The RNA was extracted using TRIzol^®^ Reagent following the manufacturer’s instructions, and the subsequent RNA-seq and RT-PCR analysis was processed as described previously (Lin et al., 2020). All gene-specific primers used in this study are listed in the supporting materials. The fold change of each gene in the experiment group (E group) compared with the control group (C group) was tested in three replicates and was calculated with reference to the internal reference genes RDN18 using the 2^−ΔΔCt^ algorithm (Wang et al., 2017).

### 2.4 Electron microscopy of *C. albicans* cells

*C. albicans* cells were incubated with X33 AMOP (MIC) at 37°C for 0, and 4 h (Ma et al., 2020). Thereafter, the cells were fixed with 2.5% glutaraldehyde and dehydrated with 50%, 60%, 70%, 80%, 90%, 95% and 100% ethanol gradients, followed by drying using 98% hexamethyldisilazane (HMDS). The samples were then coated with gold and imaged with the TESCAN vega 3 LMU scanning electron microscope (SEM).

Since the changes in internal cellular morphology can be used to evaluate the antifungal effect of X33 AMOP on *C. albicans*, the cells were incubated with X33 AMOP (MIC) in the YPD medium at 37°C for 0, 4, and 12 h (Su et al., 2020). The cells were then fixed with 2.5% glutaraldehyde and 1% osmic acid solution at 4°C. After dehydrating with 50%, 60%, 70%, 80%, 90%, 95% and 100% ethanol, the samples were treated with an embedding agent and acetone. The samples were then sliced in an ultra-thin microtome and stained with lead citrate solution, followed by a saturated solution of uranyl acetate and 50% for 10 min each. Thereafter, the stained samples were observed by transmission electron microscope (TEM).

### 2.5 Determination of alkaline phosphatase content

To explore the inhibitory mechanism of X33 AMOP against *C. albicans*, we measured the activity of alkaline phosphatase (AKP), an indicator frequently used to evaluate the damage or integrity of the cell wall. The AKP activities of the different concentrations of X33 AMOP treatment were determined using a spectrophotometer and an AKP kit (Nanjing Jiancheng Bioengineering Institute, Nanjing, China), according to the manufacturer’s instructions (Song et al., 2018). The cells were cultured with different X33 AMOP concentrations at 37°C for 8 h, and their supernatants were collected every 4 h to measure the AKP content.

### 2.6 Sorbitol assay

Sorbitol, an osmotic pressure stabilizer, was added into the medium according to a previous method, and its MIC changes were measured before and after addition (Leite et al., 2014). Thereafter, MIC and 1/2 MIC cultures were serially diluted and spread on YPD agar. The plates were incubated at 37°C for 36 h, and the number of *C. albicans* colonies on each plate was recorded.

### 2.7 Permeability of cell membranes

The cell membrane is an important physiological structure that maintains the intracellular environment stability of cells. When the cell membrane is destroyed, its barrier function is damaged, releasing the intracellular substances into the extracellular environment. Membrane permeability was measured by the FDA-PI two-color fluorescence method (Ross et al., 1989). Different concentrations of X33 AMOP and sterile water were added to the cell suspension and cultured on a shaker incubator at 37°C and 200 r/min for 12 h. The cell solution was placed on the slide and stained with 2 µL fluorescein diacetate (FDA), followed by 5 µL propidium iodide (PI) for 8 min each. The excess dye was washed off, and the cells were observed under an inverted fluorescence microscope (Nikon).

### 2.8 Determination of oxidative stress parameters in *C. albicans*

To explore the oxidative damage of *C. albicans* caused by X33 AMOP treatment, we measured the level of the reactive oxygen species (ROS), hydrogen peroxide (H_2_O_2_) and malondialdehyde (MDA) according to the previous study (Ren et al., 2020). Briefly, the cells were exposed to 1/4 MIC, 1/2 MIC and MIC of X33 AMOP for 2, 4, and 8 h. Sterile water was used as the control. The cells were resuspended in PBS to 10^6^ CFU/mL, and the ROS was examined by the reactive oxygen species assay kit (Nanjing Jiancheng Bioengineering Institute, Nanjing, China), according to the instructions. The H_2_O_2_ content of the different concentrations of X33 AMOP treatments was measured using a hydrogen peroxide kit (Nanjing Jiancheng Bioengineering Institute, Nanjing, China), according to the instructions. Conversely, the thiobarbituric acid method was used to measure the MDA content of the cells.

### 2.9 Effect of X33 AMOP on glucose-stimulated acidification of the external medium

After incubation for 10 h at 37°C on a shaker at 200 r/min, *C. albicans* cells were collected, washed, and resuspended in sterile water. Added X33 AMOP, with sterile water as the control, and then incubated at 37°C for 2 h. Then, washed with sterile water to remove the antibacterial peptide and suspended in 10% C_6_H_12_O_6_ solution, and the extracellular pH was measured at 0, 1, 2,4, and 6 h respectively (da Silva Neto et al., 2020).

### 2.10 Determination of pyruvic acid, intracellular reducing sugar, and soluble protein contents

Pyruvic acid is a crucial metabolite involved in various synthesis and catabolism pathways, including amino acid synthesis, glycolysis, gluconeogenesis pathway, and tricarboxylic acid cycle (TCA cycle). Pyruvic acid content was measured according to a previous method with slight modifications (Zou et al., 2015). Briefly, the samples were homogenized in 8% trichloroacetic acid, centrifuged at 6000 r/min for 10 min, and the supernatant was collected for pyruvic acid content determination. Reducing sugars and soluble proteins are indispensable cellular components necessary for maintaining life activities. Furthermore, soluble protein content was determined using a soluble protein detection kit (Nanjing Jiancheng Bioengineering Institute, Nanjing, China), according to the manufacturer’s instructions (Chen et al., 2020a).

### 2.11 Determination of phospholipase activity and morphology transformation

Extracellular phospholipase activities were measured using a previous method with some modifications (Srivastava et al., 2018). Briefly, 1 µL of the cells was incubated on the egg yolk solid medium at 37°C for 36 h in the absence or presence of the different concentrations of X33 AMOP. The dense white zone of precipitation around the colonies showed the presence of phospholipase, whose activity (Pz) was calculated by the formula: colony diameter/(colony diameter + precipitation zone diameter). The effect of X33 AMOP on the yeast morphology of *C. albicans* was also assessed (Priya and Pandian, 2020). Different concentrations of X33 AMOP were added to the cells, and the cultures were shaken for 4, 8 and 12 h, followed by observing their morphological changes under a light microscope (Olympus).

### 2.12 Statistical analysis

Data were presented as average ± standard deviation (SD) of the three independent experiments. One-way analysis of variance (ANOVA) and Duncan’s *post hoc* test were performed with a significant p-value of < 0.05 using the SPSS statistical software to analyze the significant difference between the values of control and treated samples.

## 3 Results

### 3.1 X33 AMOP inhibited the growth of *C. albicans*

**Figure 1:**
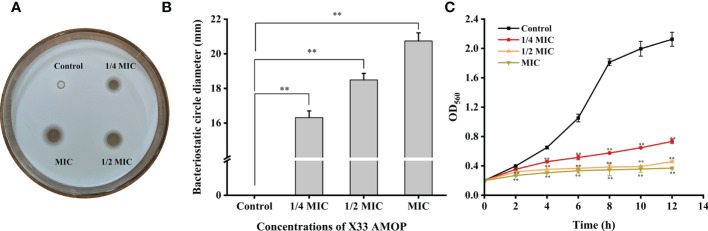
Effects of X33 AMOP on (*C*) *albicans.* **(A, B)** The diameters of the bacteriostatic circle of different X33 concentrations AMOP against (*C*) *albicans*. **(C)** The growth curves of (*C*) *albicans*. The different colors show the growth curves at different X33 AMOP concentrations. *p < 0.05, **p < 0.01.

The antimicrobial activity of X33 AMOP was performed by growth inhibition test which indicated that the MIC and MFC were 0.625 g/L and 2.5 g/L, respectively (**Table 1**). Additionally, the oxford cup test results showed that the antimicrobial diameter formed by 1/4 MIC, 1/2 MIC, and MIC of X33 AMOP were 16.31 ± 0.39, 18.49 ± 0.38, and 20.74 ± 0.47 mm, respectively (**Figures 1A, B**). Thus, the inhibitory effect of X33 AMOP against *C. albicans* increased in a dose-dependent manner. As shown in **Figure 1C**, X33 AMOP strongly inhibited the growth of *C. albicans*, and the inhibitory effect of MIC was higher than in the other groups. For the control group, *C. albicans* formed a normal growth curve and had an OD_560_ of 2.12 ± 0.10 at 12 h in the absence of X33 AMOP. However, when treated with 1/4 MIC, 1/2 MIC, and MIC of X33 AMOP, the growth of *C. albicans* exhibited a continuous decline and had OD_560_ values of 0.73 ± 0.03, 0.46 ± 0.01, and 0.37 ± 0.01 at 12 h, respectively. These results suggested a strong inhibitory effect of X33 AMOP on the growth of *C. albicans*.

**Table 1:** The MIC and MFC determination.

Concentrations (mg/mL)	20	10	5	2.5	1.25	0.625	0.3125	0.15625	0.078125	0.039	Control
Clear (-, +) a	–	–	–	–	–	–	+	+	+	+	+
Colony (-, +) b	–	–	–	+	+	+	–	–	–	–	+

a Minimum inhibitory concentration, b Minimum fungicidal concentration.

### 3.2 X33 AMOP caused cell wall damage in *C. albicans*

**Figure 2:**
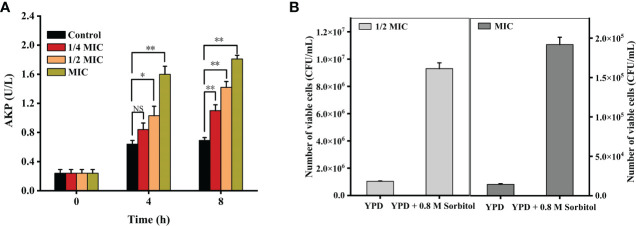
Effects of the different X33 AMOP concentrations on (*C*) *albicans* cell wall integrity. **(A)** Alkaline phosphatase activity of the different X33 AMOP concentrations on (*C*) *albicans.* The different colors show the growth of the different X33 AMOP concentrations. **(B)** The cell vitality of (*C*) *albicans.* *p < 0.05, **p < 0.01, NS: *p* > 0.05.

As shown in **Figure 2A**, the AKP activities were 0.84 ± 0.09 (*p* = 0.16 > 0.05), 1.03 ± 0.13 (*p* < 0.05), and 1.60 ± 0.11 (*p* < 0.001) U/L when the cells were treated with 1/4 MIC, 1/2 MIC, and MIC of X33 AMOP for 4 h, respectively, which were higher than that of the control group (0.64 ± 0.05 U/L). Compared with the control group, the AKP activities of 1/4 MIC, 1/2 MIC, and MIC of the X33 AMOP treatment increased by 0.59-, 1.05-, and 1.61-folds during the 8 h incubation with *C. albicans*, respectively. To further confirm this observation, we added sorbitol, a cell wall protector to the treatment groups. The logic was that the protective effect of sorbitol should be reflected by the increasing MIC values (Svetaz et al., 2007). Nonetheless, the MIC of X33 AMOP did not change when 0.8 M sorbitol was added to the incubation system, but the number of viable cells in the 1/2 MIC and MIC of X33 AMOP treatment groups reached 9.30*10^6^ ± 4.24*10^5^ and 1.92*10^5^ ± 9.31*10^3^ CFU/mL, respectively. These values were significantly higher than that of the control groups (1.04*10^6^ ± 0.28*10^5^ and 1.44*10^4^ ± 0.85*10^3^ CFU/mL) in the absence of sorbitol **Figure 2B**. Therefore, we concluded that X33 AMOP could initiate cell wall damage in *C. albicans*.

### 3.3 X33 AMOP induced membrane permeability changes in *C. albicans*

**Figure 3:**
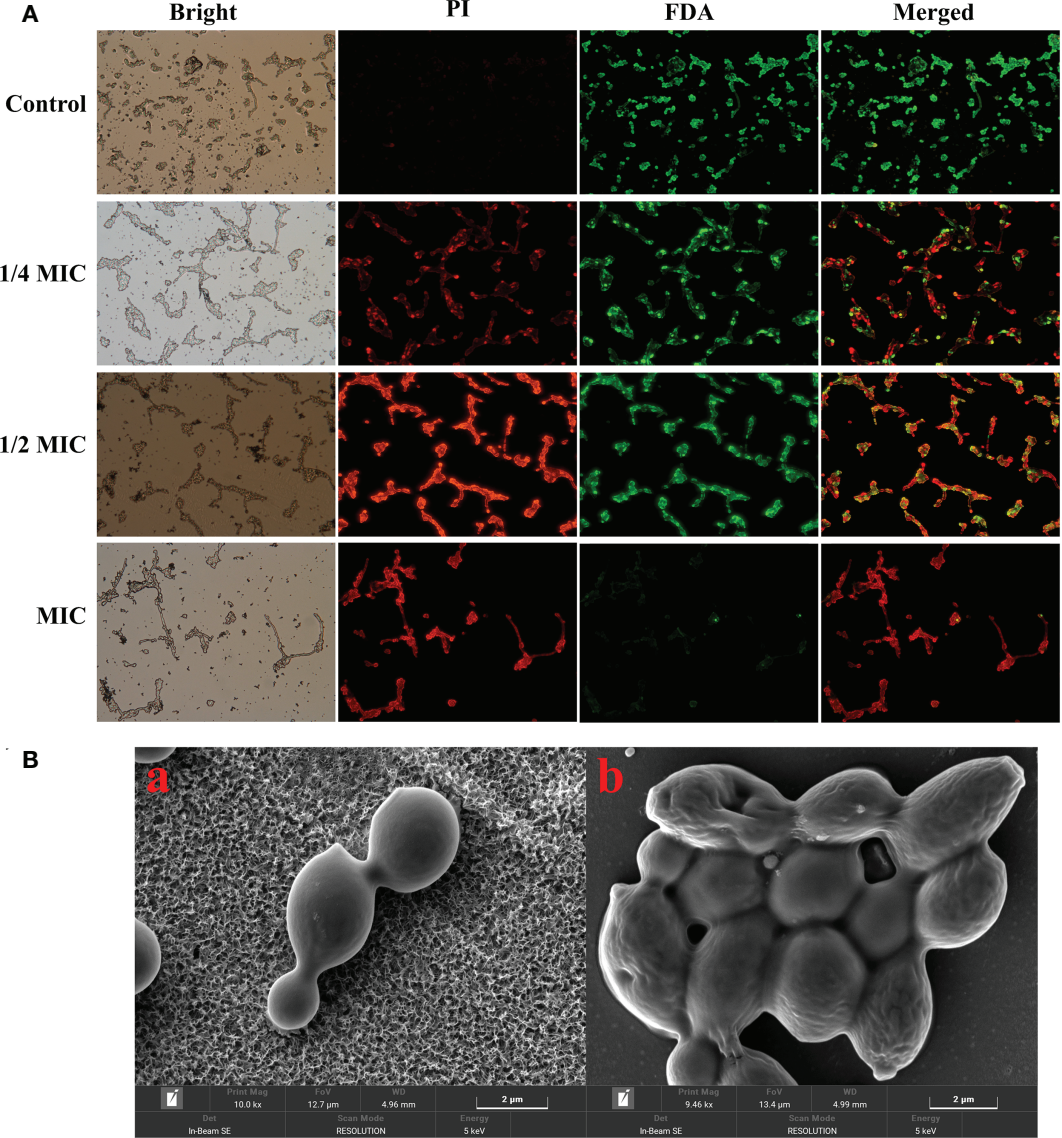
Effects of X33 AMOP on the cell membrane permeability of (*C*) *albicans*. **(A)** Fluorescent microscopy of (*C*) *albicans* cells. The green color represents viable cells, while the red color represents dead cells. **(B)** Scanning electron microphotographs of (*C*) *albicans* cells subjected to X33 AMOP treatment at the MIC for (a) 0 h, (b) 4 h.

Viable cells with integrated membrane exhibit green fluorescence, while the damaged cell membrane exhibits a red fluorescent under the fluorescence microscope. In this study, the control group cells exhibited a robust green fluorescence and a weak red fluorescence. However, the red fluorescence gradually increased, while the green fluorescence decreased after the treatment with 1/4 MIC, 1/2 MIC and MIC of X33 AMOP **Figure 3A**. Microscopy imaging of *C. albicans* using the scanning microscope indicated that the cells changed from complete, smooth, plump, and regulated morphology to deformed and wrinkled interconnection morphology after the treatment with X33 AMOP (**Figure 3B**). When the cell membrane is destroyed, macromolecular substances such as proteins, and reducing sugars, originally existing in the cell are leaked out of the cell. We found that the membrane leakage of protein reached 32.77 ± 0.41, 34.50 ± 0.77, 51.43 ± 1.82 mg/L after treatment with 1/4 MIC, 1/2 MIC, and MIC of X33 AMOP, respectively. These values were 0.45-, 0.53-, and 1.28-fold higher than that of the control group (22.56 ± 0.32 mg/L), respectively (**Figure 3C**). Similarly, the leakage of reducing sugars increased by 0.89-, 1.85- and 3.10-fold after treatment with 1/4 MIC, 1/2 MIC, and MIC of X33 AMOP, respectively (**Figure 3D**). These results showed that X33 AMOP destroyed the integrity of *C. albicans* cell membrane, resulting in the leakage of reducing sugars and proteins.

### 3.4 X33 AMOP induced oxidative stress reaction in *C. albicans*

**Figure 4:**
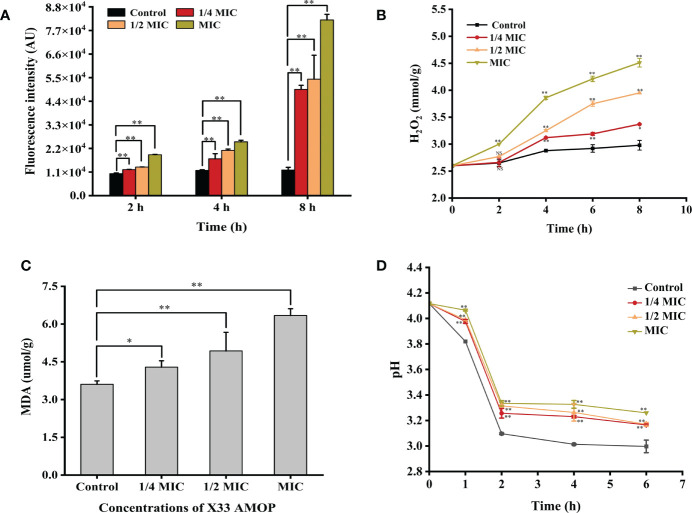
Effects of the different X33 AMOP concentrations on the oxidative stress level in (*C*) *albicans*. **(A)** The fluorescence intensity of dichlorofluorescein (fluorescence intensity is proportional to ROS). **(B)** The H_2_O_2_ contents at the different X33 AMOP concentrations. **(C)** The MDA contents at the different X33 AMOP concentrations. **(D)** Effects of X33 AMOP on the glucose-dependent acidification of (*C*) *albicans* culture medium. The different colors show different concentrations of X33 AMOP. *p < 0.05, **p < 0.01, NS: *p* > 0.05.

The accumulation of ROS has been shown to play a crucial role in programmed cell death of yeast and could therefore be an element of cell membrane damage (Sun et al., 2022). As shown in **Figure 4A**, ROS accumulation in *C. albicans* increased significantly after the treatment with X33 AMOP. After a 2 h treatment with 1/4 MIC, 1/2 MIC, and MIC of X33 AMOP, ROS accumulation in *C. albicans* increased by 0.19-, 0.30-, and 0.86-fold compared with the control group. However, the increment reached 3.14-, 3.56- and 5.88- fold when *C. albicans* cells were treated with 1/4 MIC, 1/2 MIC, and MIC of X33 AMOP for 8 h, respectively. ROS consist of superoxide anions (O_2_^-^), hydrogen peroxide (H_2_O_2_), hydroxyl radicals (OH), and nitric oxide. Lipid peroxidation promotes the formation of peroxyl radicals, which destroy the cell membrane stability, resulting in induced cell death (Patriota et al., 2016). To further explore the oxidative stress in *C. albicans*, we measured the content of H_2_O_2_. As shown in **Figure 4B**, the H_2_O_2_ contents were 3.37 ± 0.01, 3.95 ± 0.01, and 4.51 ± 0.08 mmol/g, representing 13%, 32%, and 51% increase compared to the control group (2.98 ± 0.09 mmol/g), after 8 h treatment with 1/4 MIC, 1/2 MIC, and MIC, respectively. Since ROS production damages the cell membrane, resulting in lipid peroxidation and MDA generation, we also measured the MDA contents to explore the oxidative stress effects on *C. albicans*. We found that the MDA contents in *C. albicans* were 4.29 ± 0.25, 4.93 ± 0.74, and 6.34 ± 0.27 µmol/g, which were 19%, 37%, and 76% higher than that of the control group (3.61 ± 0.14 µmol/g), under 1/4 MIC, 1/2 MIC, and MIC of X33 AMOP treatment, respectively (**Figure 4C**). Plasma membrane (PM) H ^+^-ATPase is a primary pump that plays various roles in cell metabolism and maintaining transmembrane H ^+^ electrochemical gradient (da Silva Neto et al., 2020). Upon their induction with glucose, normal cells activate the proton pump on their membranes and secrete acid to the culture medium to maintain intracellular homeostasis and osmotic pressure stability. In this study, the external medium acidification of *C. albicans* was inhibited by X33 AMOP after their induction with glucose (**Figure 4D**). When MIC of X33 AMOP was added to the medium, the pH value decreased by 0.86 after induction for 6 h, while that of the control group reduced by 1.14. This showed that the acidification ability of the control group was stronger than that of the treatment group. Thus, we concluded that X33 AMOP could induce oxidative stress in *C. albicans*.

### 3.5 X33 AMOP triggered the cellular content changes in *C. albicans*

**Figure 5:**
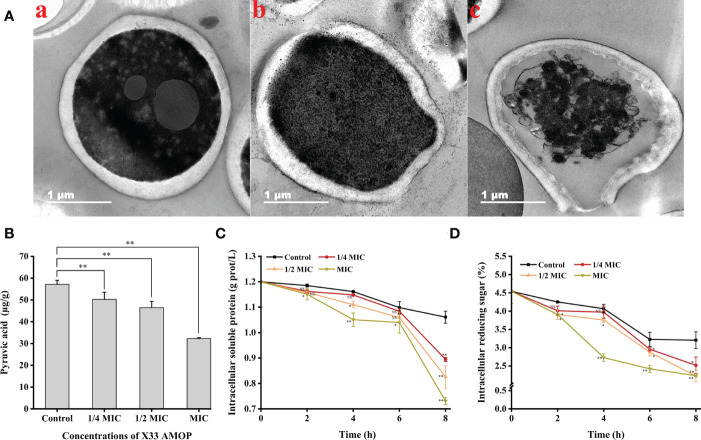
Effects of the different X33 AMOP concentrations on the cellular contents of (*C*) *albicans*. **(A)** Transmission electron microscopy of *(C) albicans* cells treated with X33 AMOP at the MIC for (a) 0 h, (b) 4 h, and (c) 12 h. **(B)** The pyruvic acid content of (*C*) *albicans* at the different X33 AMOP concentrations. **(C)** The soluble protein contents of (*C*) *albicans* at the different X33 AMOP concentrations. **(D)** The reducing sugar content of (*C*) *albicans* at the different X33 AMOP concentrations. The different colors show different concentrations of X33 AMOP. *p < 0.05, **p < 0.01, NS: *p* > 0.05.

The transmission electron microscopy results suggested that the *C. albicans* cells treated with X33 AMOP were seriously damaged, their contents were leaked out, and their organelles were agglomerated. However, the control groups exhibited dense, uniform, regular, and clearly visible cellular organelles (**Figure 5A**). What’s more, to evaluate the cellular content changes in *C. albicans* treated with X33 AMOP, we measured the intracellular pyruvic acid content using the 2,4-dinitrophenylhydrazine method. As shown in **Figure 5B**, the intracellular pyruvic acid content of the control group was 57.14 ± 1.88 µg/g, while that of the 1/4 MIC, 1/2 MIC, and MIC treatment groups were 50.25 ± 3.26, 46.46 ± 2.85, and 32.30 ± 0.38 µg/g, respectively. This showed that X33 AMOP induced metabolism disturbance in *C. albicans*. We also evaluated the intracellular soluble protein content to further evaluate the cellular content changes induced by the X33 AMOP treatment in *C. albicans*. We found that the soluble protein contents of *C. albicans* were 0.89 ± 0.01, 0.83 ± 0.04, and 0.73 ± 0.01 g/L after the 8 h treatment with 1/4 MIC, 1/2 MIC, and MIC of X33 AMOP, respectively (**Figure 5C**). These values were lower than that of the control group (1.06 ± 0.02 g/L) (**Figure 5C**). Similarly, the reducing sugar content also reduced to 2.52 ± 0.23, 2.22 ± 0.14, and 2.23 ± 0.03% when *C. albicans* were treated with 1/4 MIC, 1/2 MIC, and MIC of X33 AMOP, respectively, were lower than the control group (3.20 ± 0.23%) (**Figure 5D**). These results indicated that X33 AMOP treatment disrupts the anabolism and integrity of *C. albicans* cellular components.

### 3.6 Transcriptome analysis of *C. albicans* in response to X33 AMOP treatment

**Figure 6:**
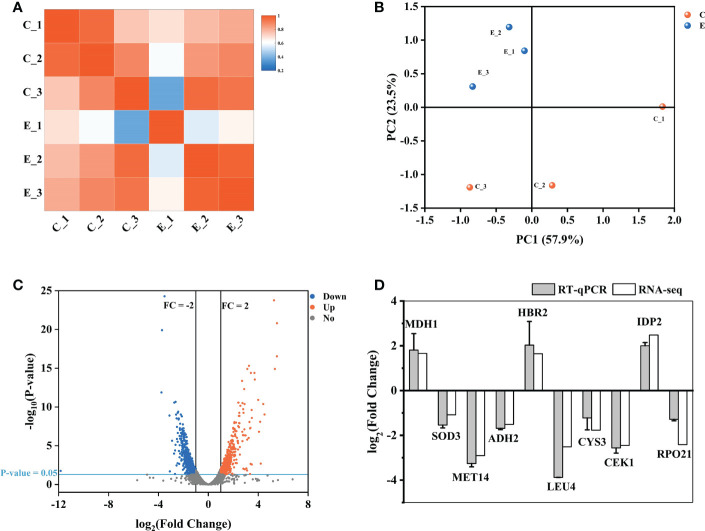
Expression analysis of different genes between the samples. **(A)** The biological repeat correlation analysis of the samples. The different colors represent correlation coefficients, and the colors closer to red indicate higher gene expression similarity among the samples. **(B)** Principal component analysis (PCA) of the samples. **(C)** A volcano plot showing the expression analysis of the different genes. The red dots represent significantly up-regulated genes, the blue dots represent significantly down-regulated genes, and the grey dots represent genes without significant expression differences. **(D)** The RT-qPCR results showing the expression of the different genes. The vertical axis represents the fold changes. Up-regulated and down-regulated genes are represented by positive and negative values, respectively.

Here, we used transcriptome analysis of gene expression to comprehensively construct the inhibitory model of X33 AMOP against *C. albicans*. The quality assessment results showed that the percentage of bases with a sequencing quality of 99% (Q20) was higher than 85%, while the sequencing quality of 99.9% (Q30) was higher than 80%. Moreover, the error rates of six samples were less than 0.1%, indicating that sequencing samples were not contaminated and met the requirements for transcriptional analysis (**Table 2**). The biological repeat correlation analysis was performed to compare the correlation of C (control group) and E (X33 AMOP treatment group) samples (**Figure 6A**). We found that the C and E samples had closer correlation values, showing a higher similarity of gene expression between the samples. The principal component analysis (PCA) reflects intra-group repeatability and inter-group difference by clustering the samples into components. The replicates of groups C and E clustered well, indicating consistency between the biological replicates. The PC1 and PC2 clusters constituted 57.9% and 23.5% of the samples, respectively (**Figure 6B**). Among the 6045 expressed genes, 1140 met the screening conditions of the differentially expressed genes (p-adjust < 0.05 & |log2FC| ≥ 1), among which 532 were up-regulated, and 608 were down-regulated (**Figure 6C**). To verify the accuracy of RNA-seq data, we selected ten DEGs randomly for validation using RT-qPCR (Primer sequences were shown in **Table S1**). Consistent with the RNA-seq results, the RT-qPCR results showed that the expression of *MDH1*, *HBR2*, and *IDP2* were upregulated, while *SOD3*, *MET14*, *ADH2*, *LEU4*, *CYS3*, *CEK1*, and *RPO21* were downregulated tests (**Figure 6D**). Thus, these results proved the reliability of the RNA-seq data.

**Table 2:** The quality of sequencing.

Samples	Raw reads (million)	Clean reads (million)	Error rate (%)	Q20 (%)	Q30 (%)	GC content (%)
C_1	43.01	42.64	0.02	98.73	95.55	37.79
C_2	43.84	43.37	0.02	98.54	95.20	38.19
C_3	43.74	43.16	0.02	98.56	95.24	38.17
E_1	43.28	42.69	0.02	98.18	94.49	37.76
E_2	44.49	44.13	0.02	98.52	95.09	36.96
E_3	44.19	43.61	0.02	98.52	95.14	36.82

**Figure 7:**
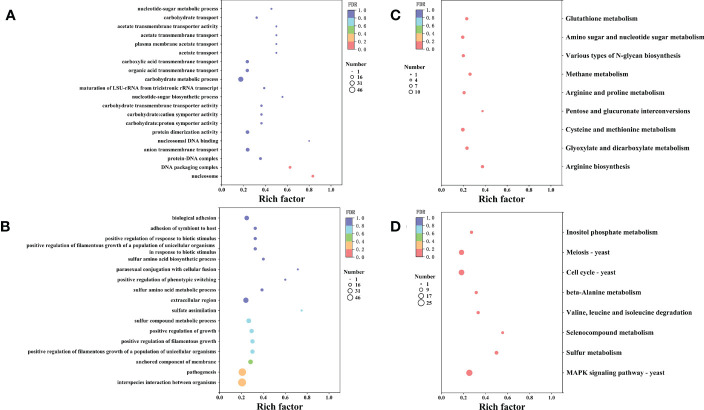
The GO and KEGG enrichment analyses of the differentially expressed genes. The vertical axis represents GO Term or KEGG Pathway, while the horizontal axis represents the enrichment factor. The dot size represents the number of genes in the GO Term or KEGG Pathway, and the dot color corresponds to the different P-values. **(A)** The top 20 GO functions of the up-regulated genes. **(B)** The top 20 GO functions of the down-regulated genes. **(C)** The top 20 KEGG pathways enrichment of the up-regulated genes. **(D)** The top 20 KEGG pathways enrichment of the up-regulated genes.

Furthermore, we conducted Gene Ontology (GO) term and KEGG enrichment analyses to systematically explore the biological functions and pathways of the obtained DEGs. The GO term analysis indicated that 518 genes were divided into three-term types and 152 hierarchies. The up-regulated genes were mainly involved in the protein, nucleic acid, and carbohydrate metabolism, while the up-regulated genes were enriched in nucleosome, DNA packaging complex and protein-DNA complex. The molecular function enrichment analysis suggested that the up-regulated genes were involved in transmembrane transporter, symporter and protein dimerization activities. Moreover, the biological process enrichment analysis suggested that the up-regulated genes were involved in transmembrane transport and nucleotide-sugar biosynthetic process (**Figure 7A**). However, the down-regulated genes were enriched in the anchored components and extracellular regions of the membrane. According to the biological process enrichment analysis, these down-regulated genes were involved in interspecies interaction between organisms, pathogenesis, sulfur compound metabolic process, and biological adhesion. Therefore, X33 AMOP induced expression changes in various genes related to various metabolism and cellular processes in *C. albicans* (**Figure 7B**). The KEGG pathway analysis results showed that 335 genes were classified into 89 pathways, among which the up-regulated genes were significantly enriched in nine (p < 0.05). Among these, the arginine biosynthesis pathway had six, while glyoxylate and dicarboxylate metabolism had seven up-regulated genes. Eight up-regulated genes were enriched in cysteine and methionine metabolism, three were enriched in pentose and glucuronate interconversions, and six were enriched in arginine and proline metabolism pathways. Among them, the top three enriched pathways were arginine biosynthesis, glyoxylate and dicarboxylate metabolism and cysteine and methionine metabolism (**Figure 7C**). The down-regulated genes were significantly enriched (p < 0.05) in eight pathways, including mitogen-activated protein kinase (MAPK) signaling, sulfur metabolism, selenocompound metabolism, valine, leucine and isoleucine degradation, beta-alanine metabolism, cell cycle-yeast, meiosis-yeast, and inositol phosphate metabolism pathways (**Figure 7D**).

### 3.7 X33 AMOP suppressed the virulence characteristics of *C. albicans*

**Figure 8:**
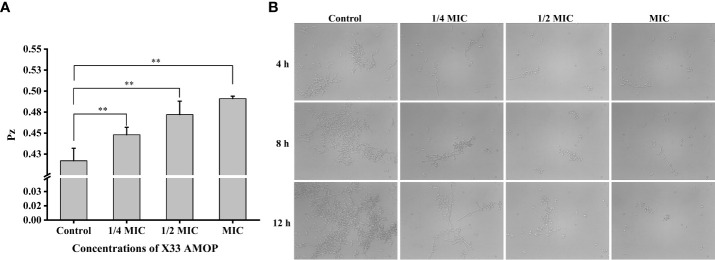
Effects of X33 AMOP on virulent attributes of (*C*) *albicans.* **(A)** The phospholipase activity values (Pz values) at the different X33 AMOP concentrations. **(B)** The transformation morphologies of (*C*) *albicans*. *p < 0.05, **p < 0.01.

The virulence factors, including adhesion, secretion of enzymes, and morphology transformation, are necessary for *C. albicans* pathogenicity (Calderone and Fonzi, 2001). Phospholipase activity was indicated by the Pz values, and higher Pz values corresponded to lower phospholipase activities. The control group had Pz value of 0.42 ± 0.02, while the Pz value of the MIC group was 0.49 ± 0.03 (**Figure 8A**), indicating that X33 AMOP could significantly reduce the phospholipase activity. Morphogenesis is a form of growth transformation from yeast and filamentous cells. The yeast cells are more conducive for reproduction, while hyphal (filamentous) ones are more suitable for invasion and virulence (Gong et al., 2019). As shown in **Figure 8B**, the transformation from yeast to hyphal cells was inhibited by X33 AMOP in a dose-dependent manner. Long and interlaced pseudo hyphae or hyphae were detected in the control group, while the X33 AMOP treatment group displayed single-yeast cells and a few filaments. These results indicated that X33 AMOP treatment could inhibit the virulence of *C. albicans*.

## 4 Discussions

In the present study, we investigated the inhibitory mechanism of X33 AMOP against *C. albicans* through transcriptome and physiological analysis. The MIC, MFC, and bacteriostatic circle diameter results showed that X33 AMOP inhibited *C. albicans* in a dose-dependent manner. Similarly, the growth curve showed that X33 AMOP significantly inhibited the growth of *C. albicans*. The number of cells, germ tubes, spores, and hypha were significantly decreased, and the *C. albicans* cells treated with X33 AMOP showed shrunken, deformed and irregular morphologies. Further morphological analysis using TEM indicated the *C. albicans* cells treated with X33 AMOP had ruptured cell boundaries, shrunken organelles, and leaked cellular contents. Thus, these phenotypic and physiological analyses illustrated that X33 AMOP has a strong inhibitory effect on *C. albicans*.

### 4.1 Cell wall integrity of *C. albicans* in response to X33 AMOP

Damage to any cell wall component can trigger various reactions, leading to the compensatory formation of another competent cell wall (Popolo et al., 2001). After the X33 AMOP treatment, the expression of genes, including *CHS1* (gene-CAALFM_C702770WA), *UTR2* (gene-CAALFM_C301730CA), *GSC1* (gene-CAALFM_C102420CA), *PHR2* (gene-CAALFM_C100220WA), and *PMT4* (gene-CAALFM_C206100WA), related to the synthesis of cell wall components was downregulated (**Table 3**). In contrast, the expression of *CHT2, CRH11, CHS7, GFA1, UAP1, GNA1, XOG1, SGA1*, and *MNN22* were upregulated after the treatment with X33 AMOP (**Table 3**). Although the chitin synthesis-related genes (*CSH1* and *UTR2*) were downregulated, the cell wall damage triggered the upregulation of cell wall related genes (*UADP1*, GFA1, *CHR11*, and *CSH7*) as compensatory response mechanisms (Lagorce et al., 2002). Interestingly, a previous study showed that when treated with micafungin, the expression of *CHT2*, *CHS1*, and *UTR2* was upregulated (Kaneko et al., 2010), contrary to our findings. The genes encoding 1,3-beta-glucan synthase (*GSC1* and *GSL2*), 1,3-beta-glucanosyltransferase (*PHR2* and *PHR3*), beta-glucan synthesis-associated protein (*ENG1*, *KRE6*, and *BGL22*), and glucan glucosidase (*XOG1*, *SGA1*, and *BGL2*) were mainly involved in beta-glucan biosynthesis. Moreover, these genes showed significant differences in their expression after the X33 AMOP treatment. Similarly, another study showed that after induction by fluconazole, the expression of *XOG1* and *BGL2*, involved in 1,3-beta-glucan transportation, were upregulated (Shao et al., 2017). In this study, we found that the X33 AMOP-induced genes encoding olichyl-phosphate-mannose-protein mannosyltransferase (*PMT1* and *PMT4*) and mannan endo-1,6-alpha-mannosidase (*DFG5*) were significantly downregulated. However, the genes encoding Alpha-1,2-mannosyltransferase (*MNN22*, *MNN23* and *MN24*) and mannan endo-1,6-alpha-mannosidase (*MNN9*) were upregulated. A previous study reported that echinocandins inhibited the synthesis of 1,3-beta-glucan, resulting in a compensatory increase of chitin content in the cell wall, which triggered the expression of mannoprotein for reshaping the cell wall (Ibe et al., 2017).

**Table 3:** Differentially expression genes in *C. albicans* after X33 AMOP treatment.

Gene ID	Gene name	Gene description	Log2FC(E/C)	Padjust	Regulate
Cell wall synthesis					
gene-CAALFM_C202010CA	CHT4	putative chitinase	-1.64	0.00	down
gene-CAALFM_C504130CA	CHT2	Cht2p	2.30	0.00	up
gene-CAALFM_C502530WA	UAP1	UDP-N-acetylglucosamine diphosphorylase	3.12	0.00	up
gene-CAALFM_C203870WA	GNA1	glucosamine 6-phosphate N-acetyltransferase	1.54	0.01	up
gene-CAALFM_C302280CA	GFA1	glutamine–fructose-6-phosphate transaminase (isomerizing)	2.79	0.00	up
gene-CAALFM_C402900CA	CRH11	transglycosylase	2.06	0.00	up
gene-CAALFM_C702770WA	CHS1	chitin synthase	-1.21	0.01	down
gene-CAALFM_C106010WA	CHS7	Chs7p	1.65	0.00	up
gene-CAALFM_C301730CA	UTR2	Utr2p	-2.28	0.00	down
gene-CAALFM_C102420CA	GSC1	1,3-beta-glucan synthase	-1.44	0.00	down
gene-CAALFM_CR00850CA	GSL2	Gsl2p	-1.35	0.00	down
gene-CAALFM_C100220WA	PHR2	1,3-beta-glucanosyltransferase	-2.73	0.00	down
gene-CAALFM_C400090WA	PHR3	1,3-beta-glucanosyltransferase	-1.33	0.02	down
gene-CAALFM_C103680WA	ENG1	endo-1,3(4)-beta-glucanase	-1.19	0.02	down
gene-CAALFM_C305830WA	KRE6	beta-glucan synthesis-associated protein	-1.21	0.01	down
gene-CAALFM_CR09420CA	BGL22	Bgl22p	-1.20	0.03	down
gene-CAALFM_C102990CA	XOG1	glucan 1,3-beta-glucosidase	2.19	0.00	up
gene-CAALFM_C301320CA	SGA1	glucan 1,4-alpha-glucosidase	1.90	0.00	up
gene-CAALFM_CR09420CA	BGL22	Bgl22p	-1.20	0.03	down
gene-CAALFM_C206100WA	PMT4	dolichyl-phosphate-mannose-protein mannosyltransferase	-1.95	0.00	down
gene-CAALFM_C702890CA	PMT1	dolichyl-phosphate-mannose-protein mannosyltransferase	-1.53	0.01	down
gene-CAALFM_C306020WA	MNN9	mannosyltransferase complex subunit	1.21	0.02	up
gene-CAALFM_C110070CA	MNN23	Mnn23p	1.40	0.02	up
gene-CAALFM_C305610WA	MNN14	Mnn14p	1.42	0.01	up
gene-CAALFM_C201300CA	MNN24	Mnn24p	1.60	0.00	up
gene-CAALFM_C110300WA	MNN12	alpha-1,3-mannosyltransferase	1.68	0.00	up
gene-CAALFM_C404770CA	MNN22	Mnn22p	2.36	0.00	up
gene-CAALFM_C200520WA	DFG5	putative mannan endo-1,6-alpha-mannosidase	-1.03	0.02	down
Cell membrane synthesis					
gene-CAALFM_C300760WA	ERG4	Delta (24(24(1)))-sterol reductase	1.22	0.01	up
gene-CAALFM_C104770CA	ERG3	C-5 sterol desaturase	1.32	0.00	up
gene-CAALFM_C108460CA	UPC2	Upc2p	-1.90	0.00	down
gene-CAALFM_C504910WA	CAALFM_C504910WA	hypothetical protein	-1.78	0.00	down
Oxidative stress					
gene-CAALFM_C200680CA	SOD5	Sod5p	-2.34	0.00	down
gene-CAALFM_C700110WA	SOD3	Sod3p	-1.08	0.03	down
energy metabolism					
gene-CAALFM_C300880WA	KGD1	alpha-ketoglutarate dehydrogenase	-1.84	0.00	down
gene-CAALFM_C403940CA	PYC2	pyruvate carboxylase 2	-1.51	0.00	down
gene-CAALFM_C701640WA	LAT1	dihydrolipoyllysine-residue acetyltransferase	-1.18	0.03	down
gene-CAALFM_C105260CA	SDH1	succinate dehydrogenase flavoprotein subunit	-1.08	0.04	down
gene-CAALFM_C401850CA	PDC12	Pdc12p	-1.19	0.04	down
gene-CAALFM_C100170WA	LEU4	2-isopropylmalate synthase	-2.51	0.00	down
gene-CAALFM_CR04530WA	FUM11	fumarase	1.03	0.01	up
gene-CAALFM_CR00540CA	MDH1	malate dehydrogenase	1.66	0.00	up
gene-CAALFM_CR02360WA	IDP2	isocitrate dehydrogenase (NADP(+))	2.48	0.00	up
gene-CAALFM_C101390CA	CAALFM_C101390CA	hypothetical protein	-1.92	0.00	down
gene-CAALFM_C404010WA	CAALFM_C404010WA	hypothetical protein	-1.08	0.00	down
gene-CAALFM_C104320WA	GPM2	Gpm2p	1.32	0.00	up
Virulence factor					
gene-CAALFM_C206170CA	ECM17	sulfite reductase (NADPH) subunit beta	-2.55	0.00	down
gene-CAALFM_C104020CA	CSH1	Csh1p	-2.04	0.00	down
gene-CAALFM_C203040WA	PLC2	Plc2p	-1.80	0.00	down
gene-CAALFM_C111590WA	PLD1	phospholipase D	-1.40	0.01	down
gene-CAALFM_C406480CA	CEK1	mitogen-activated serine/threonine-protein kinase	-2.45	0.00	down
gene-CAALFM_CR05940WA	CEK2	Cek2p	-1.96	0.00	down
gene-CAALFM_C302100WA	STE50	Ste50p	-1.63	0.00	down
gene-CAALFM_C502340CA	CST20	mitogen-activated protein kinase kinase kinase kinase	-1.33	0.00	down
gene-CAALFM_CR03900WA	HST7	mitogen-activated protein kinase kinase	-1.11	0.05	down

The extracellular AKP activities in *C. albicans* significantly increased after the X33 AMOP treatment, further confirming the cell wall disruption caused by X33 AMOP. Consistently, the peptide (C12H23O)-OOWW-NH2 (C12O3TR) and cinnamaldehyde reportedly increased the AKP activities and cell wall damage (OuYang et al., 2019; Li et al., 2020). Furthermore, the number of viable cells under X33 AMOP treatment increased with the addition of 0.8 M sorbitol, demonstrating similar effects to that of geraniol and citronellol on the cell wall of *Trichophyton rubrum* (Pereira Fde et al., 2015).

### 4.2 Cell membrane integrity of *C. albicans* in response to X33 AMOP

Ergosterol is an essential and specific component of the cell membrane responsible for adjusting the fluidity, maintaining integrity, and modulating the transportation process of the cells (Ma et al., 2020). Ergosterol biosynthesis is a complex process involving multiple genes called ERG. We found that *ERG3* (gene-CAALFM_C104770CA) and *ERG4* (gene-CAALFM_C300760WA) were upregulated in *C. albicans* after the X33 AMOP treatment. Overexpression of *ERG3*, encoding Δ^5,6^-desaturase, enhanced the synthesis of toxic steroids, casing cell damage (Sanglard et al., 2003; Prasad et al., 2019). However, upregulating *ERG4*, encoding delta (24(24(1)))-sterol reductase, which catalyzes the conversion of 5,7,22,24(28)-ergostate traenol to ergosterol, increased the ergosterol biosynthesis (Feng et al., 2017). These results indicate that X33 AMOP could interfere with the biosynthesis of cell membrane components, thus destroying its integrity. The fluorescence microscope scan, scanning electron microscope, and the increase of MDA contents also suggested that the biosynthesis and integrity of the *C. albicans* membrane were damaged by X33 AMOP. Therefore, X33 AMOP exerts inhibitory effects on protein biosynthesis and carbon metabolism by damaging the cell membrane, causing the leakage of cell contents in *C. albicans*.

### 4.3 Oxidative damage of *C. albicans* in response to X33 AMOP

The accumulation of ROS in cell mitochondria destroys various cellular components, such as protein and lipids, thus disrupting the synthesis of ATP (Wellen and Thompson, 2010). Simultaneously, cellular accumulation of ROS leads to membrane phospholipid peroxidation, which generates MDA (Su et al., 2020). In the present study, ROS significantly increased in the cells treated with X33 AMOP, suggesting that oxidative stress caused by ROS production exacerbated the cell damage of *C. albicans*. This also resulted in imbalanced redox system and increased MDA contents, indicating that X33 AMOP treatment also caused mitochondrial damage. The general mechanism of antifungal drugs against pathogenic fungi involves the accumulation of hydrogen peroxide (Delattin et al., 2014). Therefore, superoxide dismutase, which converts superoxide radicals into hydrogen peroxide, was introduced into *C. albicans* to reduce oxidative stress (Broxton and Culotta, 2016). In the present study, the expression of *SOD3* (gene-CAALFM_C700110WA), encoding iron/manganese superoxide dismutases, and *SOD5* (gene-CAALFM_C200680CA), encoding copper/zinc superoxide dismutase, were downregulated in *C. albicans* after the X33 AMOP treatment. Consistently, the expression of Cu/Zn superoxide dismutase was also downregulated in *Procambarus clarkii* under ammonia stress (Wang et al., 2020). Exposure to high H_2_O_2_ levels induced by X33 AMOP caused the cell death of *C. albicans*, indicating that X33 AMOP can cause oxidative stress in *C. albicans* and further induce cell death. Similarly, increased ROS and lipid peroxidation and upregulated expression of SODs was observed in *C. albicans* treated with honokiol (Sun et al., 2017).

### 4.4 Energy synthesis changes in *C. albicans* in response to X33 AMOP

Pyruvate is an important intermediate metabolite generated during the transformation of sugar, fatty acid, and amino acids through acetyl-CoA and TCA cycles. We found that various crucial genes, including *KGD1* (gene-CAALFM_C300880WA*)*, *PYC2* (gene-CAALFM_C403940CA), *LEU4* (gene-CAALFM_C100170WA), and *CAALFM_C101390CA* (gene-CAALFM_C101390CA), involved in pyruvate metabolism, the glycolytic pathway, and TCA cycle were downregulated. However, *MDH1* (gene-CAALFM_CR00540CA), *IDP2* (gene-CAALFM_CR02360WA), and *GPM2* (gene-CAALFM_C104320WA) were upregulated in *C. albicans* in response to X33 AMOP. The glycolytic pathway is the central metabolic pathway for carbon metabolism, which converts glucose to pyruvate and simultaneously generates ATP and NADH. The domain analysis of the hypothetical protein (encoded by *gene-CAALFM_C101390CA*) indicated its role as a transcriptional activator of glycolytic enzymes, predominantly responsible for activating the expression of glycolytic genes, which regulate the pathway (Sasaki et al., 2005). Although the expression of *GPM2*, encoding the enzyme which converts 3-phosphoglycerate to 2-phosphoglycerate, was upregulated, the transcriptional activator and the genes involved in diverting fructose 6-phosphate to the chitin synthesis pathway were downregulated. This was due to the cell wall compensation mechanism and the negative effects exerted on pyruvate biosynthesis. Several genes involved in the TCA cycle were significantly downregulated, indicating disruption of energy metabolism in *C. albicans*. Among them, *PYC2* encodes pyruvate carboxylase 2, which catalyzes the conversion of pyruvate to oxaloacetic acid, while *KGD1* encodes alpha-ketoglutarate dehydrogenase catalyzing the conversion of alpha-ketoglutarate to succinyl coenzyme A and NADP. Furthermore, the downregulation of *PYC2* and *PDC12* reduced the metabolic flux of the TCA cycle, consistent with the downregulation of HSAF in the TCA cycle and the expression glycolysis genes in *Alternaria alternate* (He et al., 2018). Downregulation of *LEU4*, encoding 2-isopropylmalate synthetase, affected pyruvate synthesis by inhibiting the conversion of 2-isopropylmalate from 2-ketoisovalerate. Furthermore, the physiological parameter analysis showed that the pyruvic acid content decreased after the X33 AMOP treatment and that the gene expression changes were consistent with the phenotype characteristics. The scanning electron microscopy showed that the organelles of the *C. albicans* treated with X33 AMOP were clustered, indicating that their functions were damaged. The intracellular pH stability is tightly controlled by the plasma membrane and vacuolar H+-ATPases (Tsang et al., 2014). Thus, the inhibition of proton extrusion greatly decreases the pH. In a present study, the extracellular medium acidification of the X33 treatment group was lower than that of the control group, indicating that the ATPase activities induced by X33 AMOP mediated the proton efflux activity of *C. albicans*. This is consistent with a previous finding showing that the pH of *C. albicans* treated with cinnamic aldehydes was higher than that of the control group (Shreaz et al., 2013). The cell wall components, soluble protein, and reducing sugar are important for cellular activities (Chen et al., 2020a). Similar to pyruvate, the protein and soluble sugar contents decreased in *C. albicans* cells treated with X33 AMOP. This demonstrates that X33 AMOP interfered with the expression of genes involved in glycolysis, TCA cycle and pyruvate metabolism, consequently hindering energy metabolism and altering the mitochondrial function of *C. albicans* cells.

### 4.5 Virulence change in *C. albicans* in response to X33 AMOP

Attenuating the virulence factors is the main inhibitory effect exhibited by antifungal drugs (Talapko et al., 2021). In this study, we found that the morphological transformation and phospholipase activity of *C. albicans* were inhibited after the X33 AMOP treatment. The expression of the hyphal formation and virulence-related genes was downregulated by approximately 1.40- to 2.45-fold. These gene included *ECM17* (gene-CAALFM_C206170CA), *CEK1* (gene-CAALFM_C406480CA), *CEK2* (gene-CAALFM_CR05940WA), *PLC2* (gene-CAALFM_C203040WA), *PLD1* (gene-CAALFM_C111590WA), and *STE50* (gene-CAALFM_C302100WA). The biosynthesis of methionine and cysteine is regulated by *ECM17*. The deficiency of these amino acids affects the expression of various proteins, disrupts the activities of signaling pathways, and interferes with the adhesion and filamentous growth in *C. albicans* (Li et al., 2013). *CEK1* and *CEK2*, encoding the extracellular signal-regulated kinase (CEK), were involved in the MAPK pathway, mainly responsible for the hyphal mating, morphological transformation, and cell wall stress adaptation in *C.albicans* (Chen et al., 2020b). Moreover, deleting the *CEK1*, *CST20*, and *HST7*, inhibited hyphal morphogenesis in *C. albicans* (Leberer et al., 1996; Csank et al., 1998). It is speculated that downregulating these genes may also affect hyphal formation. *PLD1* exhibits a high-level expression when *C. albicans* changes from yeast to hyphal growth form, probably due to the biosynthesis of phosphatidic acid and diacylglycerol (Hube et al., 2001; Dolan et al., 2004).

## 5 Conclusion

This study reveals the detailed molecular mechanisms of X33 AMOP against *C. albicans* through transcriptomic analysis and changes in physiological parameters. Our results show that X33 AMOP affects the cellular components and damages the integrity of the membranes and cell walls by stimulating oxidative stress and inducing mitochondrial dysfunction in *C. albicans*. This shows that X33 AMOP exhibits significant inhibitory effects on the virulence of *C. albicans*. Thus, this study provides possible targets for X33 AMOP against *C. albicans* and a theoretical basis for the antifungal activity of antimicrobial peptides.

## Data availability statement

The original contributions presented in the study are included in the article/**Supplementary Material**. Further inquiries can be directed to the corresponding authors.

## Author contributions

QL conducted the experiments, wrote the first draft, and revised the draft. provided investigation and experimental technique support, and performed the response of the draft. BZ and XW designed the experiments, provided guidance for the experiments, prepared the manuscript, and provided financial support for the experiments. All authors contributed to the article and approved the submitted version.
