# Supplementary material for: Physiological and transcriptome analysis of Candida albicans in response to X33 antimicrobial oligopeptide treatment
Source: Front Cell Infect Microbiol. 2023 Jan 19;13:1123393. doi: 10.3389/fcimb.2023.1123393 (PMC9892945; doi:10.3389/fcimb.2023.1123393)
Supplement: Supplementary file 1 [file Table_1.doc]

**Supplementary materials**

**Table S1.** **Primer sequences for gene amplification by qRT-PCR**

| **Gene name** | **Gene description** | **Log2FC(E/C)** | **Primer sequences** |
| --- | --- | --- | --- |
| MDH1 | malate dehydrogenase | 1.66 | F: AAAGAGTTCAATTTGGTGGCGAT  R: ACAGCAGCTAAAATTGATTCGG |
| SOD3 | Sod3p | -1.08 | F: TCGTTGCTCAGTATGGGTCTGTTTC  R: CTGATTGGCAGTAGTGACCACATCC |
| MET14 | adenylyl-sulfate kinase | -2.90 | F: TGCTCCTTATGAAGCTCCCGA  R: TGTTCAGCAGCTTGTTCAACAGA |
| ADH2 | alcohol dehydrogenase | -1.51 | F: AGCCGTTGATTTCTTCACAAGAGGT  R: GGCATGAATGCGCTTATTTGTCGTT |
| HBR2 | alanine--glyoxylate transaminase | 1.65 | F: CAATGGCAACACCTTCACAAGC  R: CCCAACCCAAAGTACCTGATC |
| LEU4 | 2-isopropylmalate synthase | -2.51 | F: AGTAATCACTAAAGCTCCCCGCTG  R: CACGCTTGGCCCCTTTTACCG |
| CEK1 | mitogen-activated serine/threonine-protein kinase | -2.45 | F: ATTGCATCGAGATTTAAAACCAC  R: AATAAAGGTCTACCACTCAACATT |
| IDP2 | isocitrate dehydrogenase (NADP (+)) | 2.48 | F: TTGGGTTTGATGACCTCAGTGTTGG  R: TGTCTAGTGACAGTACCGTGAGCAG |
| RPO21 | DNA-directed RNA polymerase II core subunit | -2.41 | F: GGTTTGGAAGCTGCTGGTATTGACC  R: ACGTTCAGCAATACCTGGGAACATG |

**Table S1. Differentially expressed genes**
